# Wound Healing and Antioxidant Properties of *Launaea procumbens* Supported by Metabolomic Profiling and Molecular Docking

**DOI:** 10.3390/antiox11112258

**Published:** 2022-11-16

**Authors:** Shaimaa R. Ahmed, Ehab M. Mostafa, Arafa Musa, Enas Ezzat Rateb, Mohammad M. Al-Sanea, Dalia H. Abu-Baih, Mahmoud A. Elrehany, Entesar Ali Saber, Mostafa E. Rateb, Usama Ramadan Abdelmohsen

**Affiliations:** 1 Department of Pharmacognosy, College of Pharmacy, Jouf University, Sakaka 72341, Saudi Arabia; 2 Department of Pharmacognosy, Faculty of Pharmacy, Cairo University, Kasr El-Ainy Street, Cairo 11562, Egypt; 3 Pharmacognosy and Medicinal Plants Department, Faculty of Pharmacy (Boys), Al-Azhar University, Cairo 11884, Egypt; 4 Department of Physiology, Faculty of Medicine, Beni-Suef University, Beni-Suef 62521, Egypt; 5 Pharmaceutical Chemistry Department, College of Pharmacy, Jouf University, Sakaka 72341, Saudi Arabia; 6 Department of Biochemistry and Molecular Biology, Faculty of Pharmacy, Deraya University, New Minia 61111, Egypt; 7 Department of Biochemistry, Faculty of Medicine, Minia University, Minia 61519, Egypt; 8 Department of Medical Sciences (Histology), Deraya University, New Minia 61111, Egypt; 9 School of Computing, Engineering & Physical Sciences, University of the West of Scotland, Paisley PA1 2BE, UK; 10 Department of Pharmacognosy, Faculty of Pharmacy, Minia University, Minia 61519, Egypt; 11 Department of Pharmacognosy, Faculty of Pharmacy, Deraya University, New Minia 61111, Egypt

**Keywords:** *Launaea procumbens*, antioxidant, wound healing, LC-HRMS profiling, docking, luteolin 8-C-glucoside

## Abstract

Wounds adversely affect people’s quality of life and have psychological, social, and economic impacts. Herbal remedies of *Launaea procumbens* (LP) are used to treat wounds. In an excision wound model, topical application of LP significantly promoted wound closure (on day 14, LP-treated animals had the highest percentages of wound closure in comparison with the other groups, as the wound was entirely closed with a closure percentage of 100%, *p* < 0.05). Histological analysis revealed a considerable rise in the number of fibroblasts, the amount of collagen, and its cross-linking in LP-treated wounds. Gene expression patterns showed significant elevation of TGF-β levels (2.1-fold change after 7 days treatment and 2.7-fold change in 14 days treatment) and downregulation of the inflammatory TNF-α and IL-1β levels in LP-treated wounds. Regarding in vitro antioxidant activity, LP extract significantly diminished the formation of H_2_O_2_ radical (IC_50_ = 171.6 μg/mL) and scavenged the superoxide radical (IC_50_ of 286.7 µg/mL), indicating antioxidant potential in a dose-dependent manner. Dereplication of the secondary metabolites using LC-HRMS resulted in the annotation of 16 metabolites. The identified compounds were docked against important wound-healing targets, including vascular endothelial growth factor (VEGF), collagen α-1, tumor necrosis factor-α (TNF-α), interleukin-1β (IL-1β), and transforming growth factor-β (TGF-β). Among dereplicated compounds, luteolin 8-C-glucoside (orientin) demonstrated binding potential to four investigated targets (VEGF, interleukin 1β, TNF-α, and collagen α-1). To conclude, *Launaea procumbens* extract could be regarded as a promising topical therapy to promote wound healing in excisional wounds, and luteolin 8-C-glucoside (orientin), one of its constituents, is a potential wound-healing drug lead.

## 1. Introduction

Wound healing problems are serious challenges facing human health. Wounds are a primary cause of physical injury [1]. Wounds, particularly of chronic nature, cause grave public health problems because they adversely affect a large number of people’s quality of life and have psychological, social, and economic impacts [2].

Wound healing is a comprehensive and sophisticated convalesced system in response to tissue damage. Optimal wound healing can be accomplished by preventing excessive growth of tissue injury while providing enough tissue perfusion, adequate oxygenation, nutrition, and a supportive protective environment that modulates the normal physiology and anatomy of the injured tissue [3]. The main objective of wound healing is restoring tissue structure, which is achieved by a series of events, including hemostasis, inflammation, granulation, re-epithelialization, extracellular matrix remodeling, and maturation [4].

Initially, clotting factors are released to stop bleeding. Rapidly after homeostasis, the inflammation stage starts, in which monocytes, neutrophils, and macrophages migrate to the wound site. Granulation of tissue occurs by myofibroblast and fibroblast regeneration of collagen fibers, blood vessels, and lymphatic vessels [3]. Keratinocytes approach the granulation tissue to start the re-epithelialization. The dermal fibroblast cell population actively remodels the granulation tissue’s disorganized extracellular matrix (ECM) as the wound matures [5].

Reactive oxygen species (ROS) and increased oxidative stress contribute significantly to controlling the proper healing process by promoting inflammation, hemostasis, angiogenesis, granulation tissue formation, extracellular matrix formation, wound closure, and maturation [6]. Antioxidants are supposed to hasten wound healing by alleviating wound oxidative stress. They are essential in controlling the harm that ROS may cause to biological components such as DNA, protein, and lipids [7,8]. On the other hand, a wound area with high levels of ROS can induce collagen deterioration and consequent ECM damage. When the ECM is damaged, processes essential for wound healing, including angiogenesis and re-epithelization, are suppressed [9,10]. In addition, increased ROS can cause inflammation, boost inflammatory cytokines, and subsequently prolong inflammation [11].

Transforming growth factor-β (TGF-β) is a molecular player in several cellular processes, including wound healing pathways [12]. TGF-β is rapidly upregulated and released following acute damage. TGF-β is required for the initiation of inflammation and the creation of granulation tissue. Furthermore, it promotes angiogenesis, keratinocyte migration, and wound contraction [13,14]. Additionally, TGF-B plays a key role in fibrosis by upregulating ECM formation (mainly inducing collagen production by fibroblast); thus, an excessive presence of this cytokine can lead to skin fibrosis [15]. Proinflammatory cytokines such as interleukin-1β (IL-1β) and tumor necrosis factor-α (TNF-α) are among the first factors released in response to skin injury. They govern immune cell activities during regeneration and impact keratinocytes and fibroblasts [16]. Inflammatory cytokines may be crucial in the early phases of wound healing because they promote leukocyte recruitment [17].

Due to the unavoidable adverse effects of current pharmaceutical products for wound treatment, it is crucial to identify an alternative treatment with fewer adverse effects and greater efficacies [18]. Therefore, urgent attention is mandatory for the development of new drugs to promote the wound healing process.

Plants are effective wound healers, and they naturally repair wounds. The discovery of cellular mechanisms of medicinal plants with wound healing potential may be useful in clinical practice and the development of new healing medications for human use [19]. Owing to the presence of secondary metabolites of various classes, researchers have identified traditional folklore medicines and their extracts as a valuable option for wound therapy during the last few decades [20].

Plants in the genus *Launaea* (Asteraceae family) are used in ethnomedicine for the treatment of wounds [2]. *Launaea* is regarded as a source of diverse secondary metabolites, including sesquiterpenoid, flavonoid, and phenolic compounds that display relevant biological activities. Members of this genus are reported to have significant biological activities, such as antibacterial, antioxidant, cytotoxic, and anti-inflammatory activities [21]. 

Different *Launaea* species have been utilized for wound healing properties. For example, *Launaea spinosa* was reported to have anti-inflammatory and antiangiogenic activity via modulating the release of inflammatory cytokines [22]. Additionally, *Launaea spinosa* experienced a strong antioxidant and antibacterial activity, as reported by Asif Saadullah, which contribute to the wound healing activity [22]. In a case study, topical treatment with *Launaea nudicaulis* for 28 days resulted in the complete healing of a chronic nonhealing wound with no side effects [23].

*Launaea procumbens* (LP) is a well-reported source of herbal medicine used traditionally for various purposes as a treatment for skin problems, tumors, and dysentery [24]. Ayurvedic and herbal remedies of this plant are employed in wound healing [25], painful urination, and reproductive diseases [26]. Additionally, the plant has been reported to have cytotoxic properties [24], antimicrobial activity [27], antiurolithiatic activity [28], protect kidneys from CCl_4_-induced nephrotoxicity [29], and protect lungs against CCl_4_-induced pulmonary damages [30]. Phytochemical screening showed the presence of flavonoids, phenols, tannins, alkaloids, and coumarins [31]. Phytochemical study of LP by GC/MS revealed the presence 1-H-pyrazole, β-amyrin, α-amyrin, lupeol, 1-H-imidazole, D-glucose, inositol, and fructose in different fractions [24]. 

Despite the plethora of studies describing the immense therapeutic relevance of *Launaea procumbens*, there is no scientific proof regarding the wound healing properties of this species, and nothing is traced about its metabolite profile.

In this context, this work was conducted to explore the in vivo wound healing potential of *Launaea procumbens* using an excision wound model in rabbits, emphasing the underlying molecular mechanism and its in vitro antioxidant activity. Furthermore, secondary metabolites profiling of the plant extract by LC-HRMS was carried out. The identified compounds were then docked against important wound-healing targets such as collagen α-1, vascular endothelial growth factor (VEGF), TNF-α, TGF-β, and IL-1β to explore the chemical compounds involved in the wound healing activity. According to the literature review, this study is the first thorough description of LP secondary metabolites profile and wound healing potential.

## 2. Materials and Methods

### 2.1. Plant Material and Extraction

*Launaea procumbens* aerial parts were collected in March 2019 from the Bosita area, Al-Jouf, Saudi Arabia. Mr. Hamedan Al-Ogereef, of the Camel and Range Research Center, Jouf, KSA, verified its authenticity. A voucher specimen (Mor-3-2020) was kept at the Pharmacognosy Department, Faculty of Pharmacy, Deraya University, Egypt, for future reference.

Dried and powdered aerial parts (4 kg) were extracted five times with methanol (8 L) at room temperature using ultraturrex homogenizer followed by evaporation under vacuum at 60 °C using rotary evaporator to obtain 300 g *Launaea procumbens* (LP) extract (7.5% yield). The methanolic extract was lyophilized and kept at −20 °C for preparation of gel as a pharmaceutical formulation.

### 2.2. Chemicals and Reagents

Acetonitrile, methanol, and water of HPLC grade (Thermo Fisher Scientific Inc., Dublin, Ireland) were used for LC-HRMS analysis. Other chemicals of analytical grade used in the current study were provided from Sigma-Aldrich Chemical Co. (Wicklow, Ireland).

### 2.3. Animals

This study was approved by the ethical review board of the faculty of pharmacy, Deraya University (approval number: 3/2021), which specified that animals must not suffer during the experiments and should be treated in accordance with the declaration of Helsinki. A total of 18 adult male New Zealand Dutch albino rabbits (8 months old) weighing 2.0–2.5 kg were used in this investigation. Animals were housed in polypropylene cages and had complete access to a standard pellet diet and water. Animals were acclimatized to the standard laboratory conditions of the well-ventilated housing seven days before the investigation started, with relative humidity (44–56%) and light and dark cycles (12:12 h) at a constant temperature (25 ± 2 °C).

### 2.4. Preparation of Test Sample Topical Gel

Lyophilized LP was prepared by dissolving *Launaea procumbens* dry extract in vehicle (2 g/100 mL) and kept at 4 °C in the dark. Each test extract was applied topically to the wound site as soon as it was prepared.

### 2.5. In Vivo Wound Healing

According to the procedure outlined by Tramontina et al., 2002 [32], to evaluate the wound healing impact of LP extract, an excision wound model was applied. The rabbits were anesthetized using 0.01 mL Ketalar^®^ (Ketalar, Sankyo Lifetech Co., Ltd., Tokyo, Japan) injected intraperitoneally (I.P.). The back of animals was shaved, and then a circular excision wound of 6 mm diameter was induced on the back of each rat by a biopsy punch within the shaved area [32]. During the whole experiment, the wounds were not dressed.

Three groups of six rabbits each were included in this study, making a total of eighteen rabbits. Group 1 (control group): only a blank vehicle was used to treat the wound; Group 2: 2% LP extract was used to treat the wounds; Group 3: (MEBO) wounds were treated with MEBO ointment (Julphar Gulf Pharmaceutical Industries, Ras Al Khaimah, United Arab Emirates) as a comparable control drug.

All treatments of 0.5 g were applied topically on the wound twice daily for fourteen days until the wounds had mostly healed. Photographs were taken every 3 days using a camera (Fuji, S20 Pro, Sendai, Japan) posing vertically to middle of the wound with a distance of 6 cm to visually monitor the gross morphological changes in the wound area until the excision wounds were fully cured [33]. Subsequently, the percentage wound closure was calculated using the following formula:

$$\frac{\left(\;wound\;area\;on\;day\;0-wound\;area\;of\;on\;specific\;day\right)\times 100}{Area\;of\;wound\;on\;day\;0}$$

where *n* is the number of days (3rd, 7th, 10th, and 14th).

Seven and fourteen days after wound induction, full thickness tissue samples were harvested from the wounds under anesthesia for further analysis of gene expression and the histological study.

### 2.6. Histological Analysis

Wound samples from dorsal surface were collected, fixed in 10% buffered formalin, and then treated with a graded series of xylene and alcohol before paraffin embedding. Using a microtome, tissue slices were cut into 4 µm thicknesses, and they were then stained with hematoxylin and eosin. Additionally, Masson trichome staining was performed to evaluate the collagen deposition [34,35].

### 2.7. Gene Expression Data Analysis

The ultrasonic homogenizer was used to homogenize approximately 50 mg of dorsal wound tissue (Sonics-Vibracell, Sonics and Materials Inc., Newtown, Fairfield County, CT, USA) in 0.5 mL TRIzol TM reagent (Invitrogen-ThermoFisher Products & Kits, Amresco, LLC-Solon, Waltham, MA, USA). Following the manufacturer’s directions, total RNA was isolated from tissues. NanoDrop 1000 (Thermo Scientific, Waltham, MA, USA) was used according to the ratio of A260/A280 to spectrophotometrically measure the concentration and the purity of the extracted RNA [36]. Samples with an acceptable purity were used for reverse transcription using high-capacity reverse transcription kit (Thermo Scientific, Waltham, MA, USA) and oligo-dT primers. Transcripts’ expression of IL-1β, TGF-β, and TNF-α (Table S1) was detected by quantitative RT-PCR. The SYBR Green PCR Master Mix (Thermo Scientific, Waltham, MA, USA) was used for amplification in a step-one real-time PCR thermal cycler (Applied Biosystems, Thermo Fischer Scientific, Waltham, MA, USA) following the manufacturer’s instructions. After calibration to GAPDH as a housekeeping gene, the relative gene expression was determined using the comparative CT (Threshold Cycle) method [37].

### 2.8. In Vitro Antioxidant Activity

The antioxidant potential of LP methanolic extract was assessed by two methods, hydrogen peroxide (H_2_O_2_) scavenging activity [38] and superoxide anion scavenging activity [39]. For positive control, ascorbic acid was used.

### 2.9. Statistical Analysis

Results (*n* = 6) were presented as mean ± standard deviation (S.D). Dunnett’s test was used following one-way analysis of variance (ANOVA). For statistical calculations, Graph Pad Prism 7 was used (Graph pad Software, San Diego, CA, USA). *p*-values less than 0.05 in comparison to the control group were regarded as significant.

### 2.10. LC-HRMS Metabolomic Analysis

Profiling of the methanolic extract of LP was performed according to a previously described method [40] on an Acquity UPLC/Synapt G2 HDMS quadrupole time-of-flight hybrid mass spectrometer (Waters, MI, USA). The Dictionary of Natural Products served as the database for the identification of substances.

### 2.11. Molecular Docking

Crystal structures of IL-1β (PDB ID: 6Y8M, resolution 1.9 Å), TNF-α (PDB ID: 2AZ5, resolution: 2.10 Å), VEGF (PDB ID: 1FLT, resolution: 1.70 Å), collagen α-1 (I) chain (PDB ID: 1Q7D, resolution: 1.80 Å), and TGF-β receptor I (TGF-β R1) kinase (PDB ID: 6B8Y, resolution: 1.65 Å) were obtained from Protein Data Bank [41]. AutoDockTools [42] was used to generate the input files of identified chemical compounds, protein structures, and cocrystallized ligands. All ligands were processed with OpenBable v2.4 [43]. AutoDock Vina [44] was used for molecular docking of chemical compounds to the investigated proteins. Grid boxes were chosen, with sizes not greater than 27,000 Å3, and “exhaustiveness” was adjusted at 32, which is the recommended value in the case of employing small boxes. For crystal structures of proteins in complexes with binding molecules, cocrystallized ligands were redocked to validate the docking methodology. The interactions of docked compounds with relevant proteins were visualized and analyzed using Discovery Studio.

## 3. Results

### 3.1. Wound Contraction

**Figure 1:**
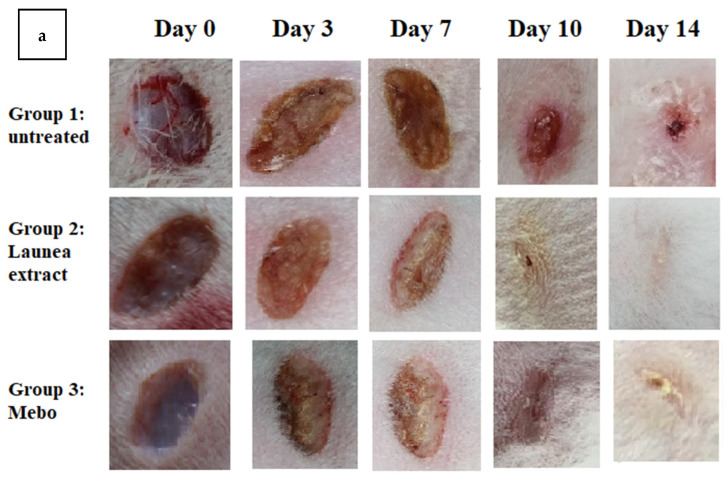

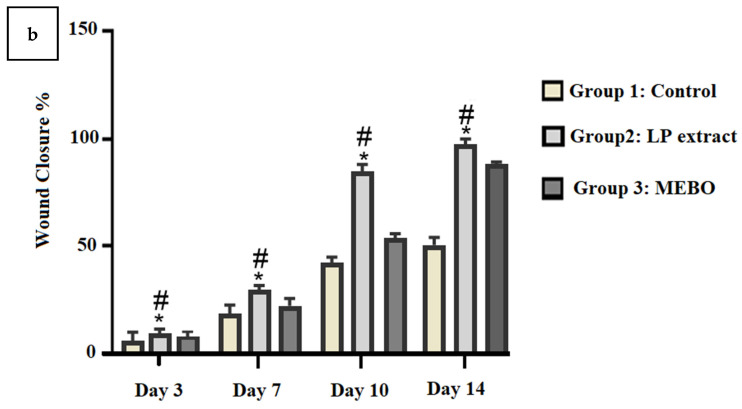
Monitoring of wound closure over 14 days in each group, (**a**). Wound closure percent in different groups, (**b**). The wound closure rate was calculated using Image J software at different times (0, 3, 7, 10, and 14 days) after wound. Values were presented as means ± S.D, with * *p* < 0.05 LP group in comparison with control group and # *p* < 0.05 LP group in comparison with MEBO^®^ group.

Figure 1a showed the wound healing in control, LP-treated, and MEBO^®^-treated groups. Rabbits in group 1 showed impaired wound healing. Further, based on calculations of percent closure rate (Figure 1b) calculated at different time intervals, LP treatment improved wound healing in rabbits compared with the control group as well as the MEBO^®^-treated group. From the third day forward, this effect was obvious (closure rate 10% for LP, 7.5% for MEBO, and 6% for vehicle-treated wounds). On day 14, LP-treated animals (group 2) had the highest percentages (rates) of wound closure in comparison with the other groups, as the wound was entirely closed, with a closure percentage of 100% (Figure 1b), while it was 88% for MEBO^®^-treated wounds.

### 3.2. Histological Study

**Figure 2:**
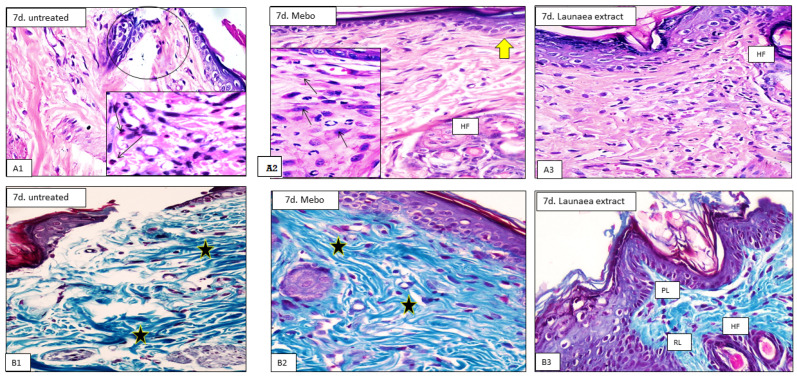
Representative photomicrograph of wound tissue on day 7 (H&E, (**A1**–**A3**) and Masson trichrome stains (**B1**–**B3**) × 200 and 400). Untreated group (**A1**) showing the wound area (circle) and the underlying sloughed granulation tissue showing congested blood capillaries, neovascularization, and inflammatory cells infiltration of mainly neutrophils (arrows in the inset). (**B1**) Showing the wound bed is loaded with a dense granulation tissue composed of compact disorganized collagen (stars). Mebo-treated group (**A2**) showing epidermis form 2–4 layers closing the wound (arrow), and the dermis showing prominent fibroblasts (arrows in the inset) and newly formed hair follicles (HF). (**B2**) Showing well-formed dermal coarse and wavy collagen bundles (stars). Launaea extract-treated group (**A3**) showing normal epidermis (arrow) with newly formed hair follicles (HF). (**B3**) Showing collagen bundles in the papillary layer, which appear as fine interlacing bundles (PL), and in the reticular layer, which appear as coarse wavy bundles (RL), hair follicles, and sebaceous glands (HF).

On day 7, histological analysis of tissue samples revealed that the control group had a normal wound edge with a normal epidermis, well-formed dermal collagen bundles, normal hair follicles, and sebaceous glands. Contrarily, blood clots that filled the wound, sloughed granulation tissue with collagen fibers densely packed in an uneven pattern, extravasated RBCs, and inflammatory cellular infiltration were seen. In the deepest part of the lesion (Figure 2(A1,B1)), the striated muscle exhibited necrotic myofibers. LP-treated wounds showed nearly the same features observed in the previous group. The epidermis is formed of 3–4 cell layers without keratin. The papillary dermis containing newly formed hair follicles and collagen bundles appeared discontinuous, while that of the reticular dermis appeared more cellular, with mainly well-developed fibroblasts (Figure 2(A3,B3)). The MEBO^®^-treated group revealed that scar tissue seals the wound and creeping epidermal cells at the wound’s margins show incomplete re-epithelization. There was a significant inflammatory cell infiltration (mostly macrophages), and collagen fibers were found filling the deformity in a reticular pattern with spacing virtually identical to that of the surrounding normal dermis. The reticular dermis was characterized by the presence of many spindle-shaped elongated fibroblasts with basophilic cytoplasm and open face oval nuclei (Figure 2(A2,B2)).

**Figure 3:**
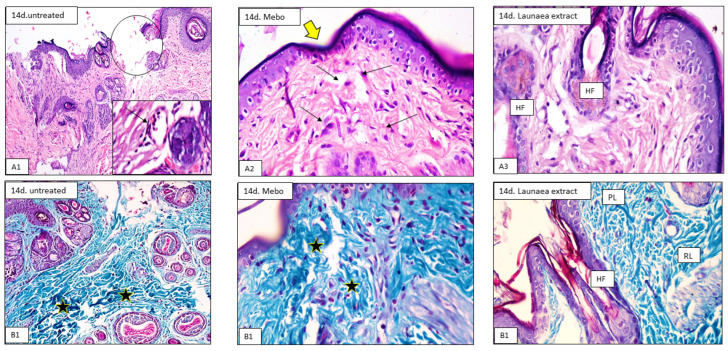
Representative photomicrograph of wound tissue on day 7 and 14 (H&E (**A1**–**A3**) and Masson trichrome stains (**B1**–**B3**) × 200 and 400). Untreated group (**A1**) showing the wide wound area (circle), and the underlying sloughed granulation tissue showing congested blood capillaries, neovascularization, and inflammatory cells infiltration of mainly neutrophils (arrow in the inset). (**B1**) Showing the wound bed is loaded with a dense granulation tissue composed of compact disorganized collagen (stars). Mebo-treated group (**A2**) showing creeping of epidermal cells closing the wound (yellow arrow), granulation tissue (stars), and inflammatory cellular infiltration of mainly macrophages (black arrows). (**B2**) Showing well-formed dermal coarse and wavy collagen bundles (stars). Launaea extract-treated group (**A3**) showing normal epidermis with newly formed hair follicles (HF). (**B3**) Showing collagen bundles in the papillary layer, which appear as fine interlacing bundles (PL), and in the reticular layer, which appear as coarse wavy bundles (RL), hair follicles, and sebaceous glands (HF).

The tissue samples taken on day 14 revealed that the wound area in the control group appeared larger and packed with thick granulation tissue which was composed of many layers of connective tissue in an acidophilic matrix and overlying massive inflammatory cell infiltration. The dermis was made up of disordered thin collagen with significant neovascularization (Figure 3(A1,B1)). However, LP-treated group showed that the skin defect covered by keratinized epidermis was formed of many cell layers. The cellular content of the dermal matrix was not pronounced and obscured by higher contents of collagen bundles (Figure 3(A3,B3)). Moreover, the MEBO^®^-treated group revealed typical stratified squamous keratinized epithelium in the skin tissue. Thin scar tissue may extend into the dermis. The dermal matrix showed several hair follicles, blood capillaries, and the absence of inflammatory cell infiltration. Collagen bundles appeared as fine interlacing bundles in the papillary dermis and as coarse wavy bundles arranged in different directions in the reticular dermis (Figure 3(A2,B2)).

### 3.3. Gene Expression Data Analysis

**Figure 4:**
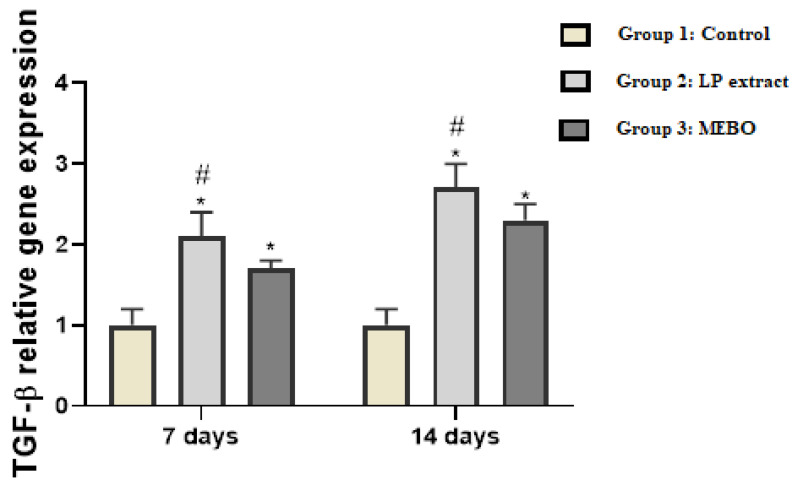
TGF-β expression in wound tissues from various groups was measured using quantitative RT-PCR on days 7 and 14. The data indicate the fold change in expression relative to the control group. The bars reflect the mean ± SD. A one-way ANOVA test is used to determine whether there is a significant difference between groups: * *p* < 0.05 LP group compared to the control group, # *p* < 0.05 LP group compared to the MEBO^®^ group.

The assessment of TGF-β gene expression using quantitative RT-PCR revealed that treatment of wounds with LP extract and MEBO^®^ showed a significant increment in TGF-β gene expression in a time-dependent behavior to reach a 2.1- and 1.7-fold change after 7 days treatment and a 2.7- and 2.3-fold change in 14 days treatment, respectively, compared with the untreated group (*p* < 0.05) (Figure 4). Furthermore, the results revealed a substantial difference (*p* < 0.05) between MEBO^®^-treated animals and LP-treated animals, with the highest expression pattern in LP-treated animals.

**Figure 5:**
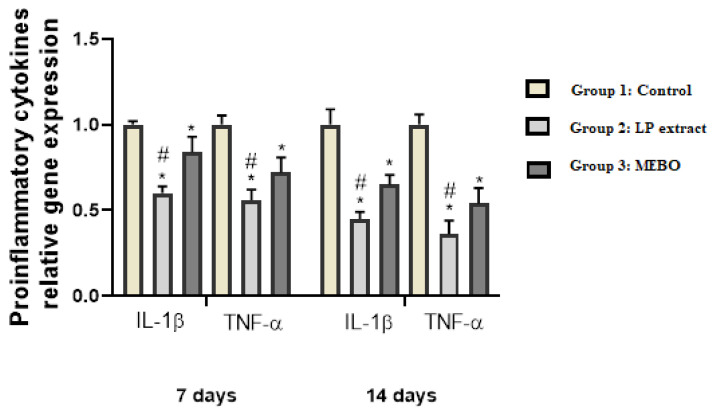
Inflammatory cytokines (IL-1β and TNF-α) expression in wound tissues from various groups were measured using quantitative RT-PCR on days 7 and 14. The data indicate the fold change in expression relative to the control group. The bars reflect the mean ± SD. A one-way ANOVA test is used to determine whether there is a significant difference between groups: * *p* < 0.05 LP group compared to the control group, # *p* < 0.05 LP group compared to the MEBO^®^ group.

On days 7 and 14, mRNA expression of wound samples (Figure 5) demonstrated that the activity of inflammatory indicators such as TNF-α and IL-1β was considerably (*p* < 0.05) suppressed in wounds treated with LP extract or MEBO^®^ in contrast to the untreated wounds. Interestingly, compared to MEBO^®^ ointment, injured rabbits treated with LP extract demonstrated a substantially greater reduction in inflammatory markers (TNF-α and IL-1β) (*p* < 0.05).

### 3.4. In Vitro Antioxidant Activity

**Figure 6:**
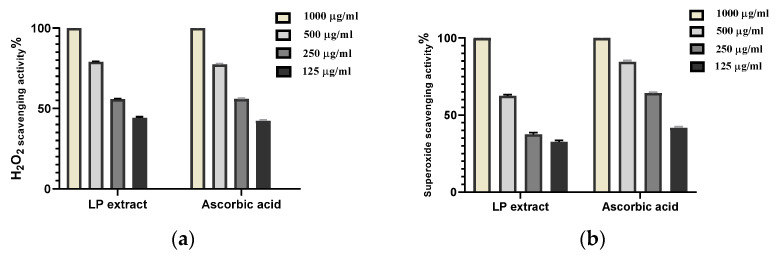
In vitro antioxidant activity of *LP* extract: (**a**) H_2_O_2_ radical scavenging activity; (**b**) superoxide radical scavenging activity.

In this investigation, LP extract was assessed for its antioxidant activity as a potential H_2_O_2_ and superoxide scavenger. The data showed that LP extract significantly inhibited the production of H_2_O_2_ free radicals (Figure 6a) in a dose-dependent way, with IC_50_ = 171.6 μg/mL. Moreover, the superoxide radical was notably scavenged using LP (Figure 6b) in a dose-dependent way, showing IC_50_ of 286.7 µg/mL, while the known ascorbic acid (positive control) showed IC_50_ = 174.2 μg/mL for H_2_O_2_ scavenging activity and IC_50_ = 164 μg/mL for superoxide scavenging activity.

### 3.5. LC-HRMS Metabolomic Analysis

**Figure 7:**
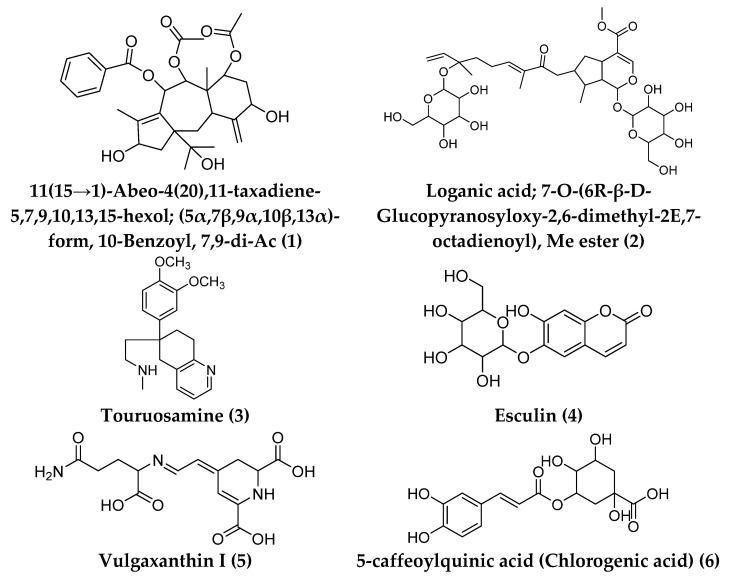

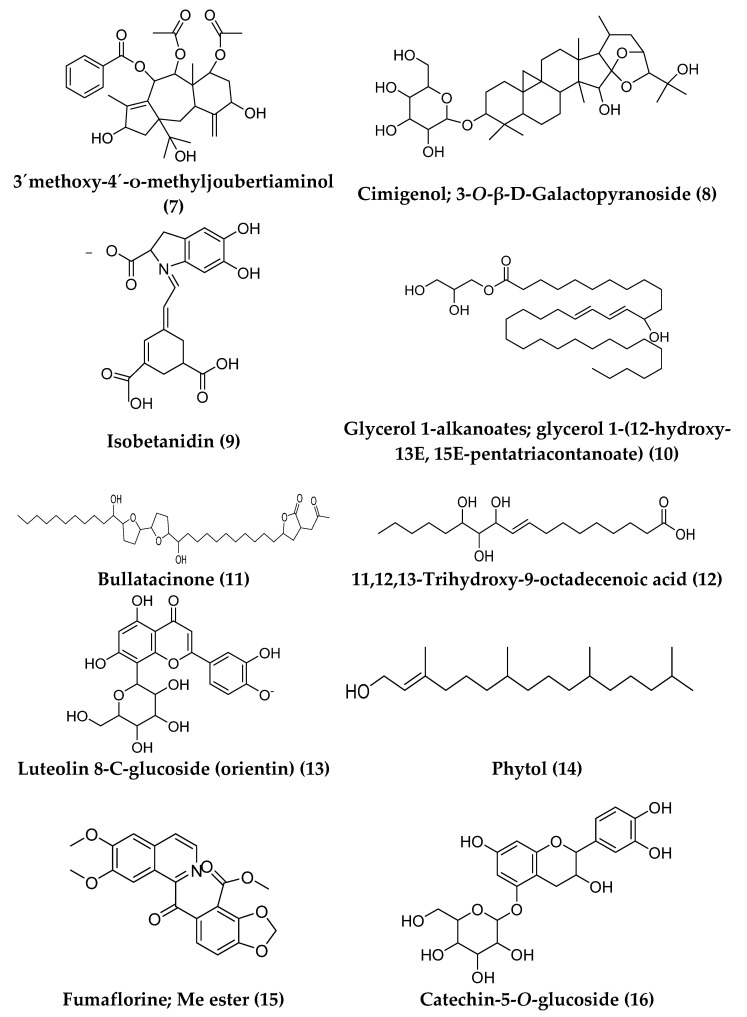
Chemical structure of the dereplicated compounds from the methanolic extract of *Launaea procambens*.

Dereplication of the secondary metabolites resulted in the annotation of 16 different compounds from the methanolic extract of LP (Figure 7). The majority of the detected compounds were found in the negative mode. LC-HRMS analysis revealed the existence of phenolic compounds, flavonoids, alkaloids, diterpenes, triterpenes, iridoid glycosides, coumarins, betaxanthins, acetogenins, and fatty acids (Table S2).

### 3.6. Molecular Docking Study

Docking studies revealed that in case of collagen α-1 (I) chain, luteolin 8-C-glucoside (orientin) demonstrated the lowest among the tested compounds’ binding energy (−6.7 kcal/mol), Table S3. For VEGF, several compounds, including luteolin-8-C-glucoside, showed close values of binding energies (Table S4). In TNF-α protein (Table S5), none of the studied compounds exceeded the reference ligand in terms of the values of predicted binding energies, but three compounds (Luteolin 8-C-glucoside (orientin), 11(15 → 1)-Abeo-4(20),11-taxadiene-5,7,9,10,13,15-hexol; (5α,7β,9α,10β,13α)-form, 10-Benzoyl, 7,9-di-Ac, and Cimigenol_3-O-ß-D-Galactopyranoside) demonstrated close values (−8.5 kcal/mol). For the TGF-β, none of the studied compounds showed lower binding energies compared to the reference ligand, with catechin-5-O-glucoside and luteolin 8-C-glucoside (orientin) being the closest in terms of binding energy (Table S6). For IL-1β protein (Table S7), multiple compounds showed lower binding energies compared to the reference ligand (−4.8 kcal/mol), with luteolin-8-C-glucoside (orientin) and 11(15 → 1)-Abeo-4(20),11-taxadiene-5,7,9,10,13,15-hexol; (5α,7β,9α,10β,13α)-form, 10-Benzoyl, 7,9-di-Ac (−6.4 and −6.3 kcal/mol, respectively) being at the top of the ranked list. 

**Figure 8:**
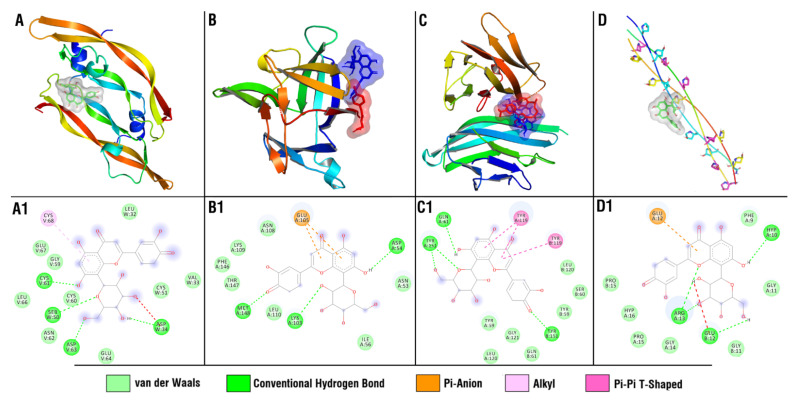
Binding modes and interaction types of the luteolin 8-C-glucoside (orientin) with the VEGF (**A**,**A1**), IL-1β (**B**,**B1**), TNF-α (**C**,**C1**), and collagen α-1 (I) chain (**D**,**D1**). In (**B**,**C**), luteolin 8-C-glucoside is colored in blue, and cocrystallized ligands of corresponding proteins are colored in red.

As a result of the performed molecular docking study, multiple compounds demonstrated binding potential to the investigated molecular targets. However, among all studied compounds, luteolin 8-C-glucoside (orientin) exceptionally demonstrated binding potential to four out of the five investigated proteins (Figure 8) and was of special interest for further analysis, posing it as a potential wound-healing drug lead.

Luteolin 8-C-glucoside (orientin) had four hydrogen bonds with ASP W 34, SER W 50, CYS V 61, and ASP V 63 amino acid residues of the VEGF, and four hydrogen bonds with the HYP A 10, GLU B 12, and ARG A 13 amino acid residues of the collagen α-1 (I) chain. Luteolin 8-C-glucoside had three hydrogen bonds with ASP A 54, LYS A 103, and MET A 148 amino acid residues of the IL-1β, while cocrystallized ligand (4-[(5-bromopyridin-2-yl)amino]-4-oxobutanoic acid) had three hydrogen bonds with MET A 148 and GLN A 149. Luteolin 8-C-glucoside (orientin) had four hydrogen bonds with GLN A 61, TYR A 151, and TYR B 151 amino acid residues of the TNF-α, while cocrystallized ligand (6,7-dimethyl-3-[(methyl{2-[methyl({1-[3-(trifluoromethyl) phenyl]-1h-indol-3-yl} methyl) amino] ethyl} amino) methyl] -4h chromen-4-one) binds mostly through Van der Waals interactions.

## 4. Discussion

In response to damaged tissue, wound healing leads to the restoration of tissue integrity. Therefore, the process of healing a wound is strongly influenced by its closure. These processes are a strong determinant of keratinocyte differentiation, granulation, re-epithelialization, and angiogenesis and proliferation, and thus a good indicator of the healing process in open wounds [45]. It is noteworthy to report that LP extract showed potent healing effects when compared to the commercially available control (MEBO^®^), as at the end of the experiment (14 days), the closure rate was 100% for LP- and 88% for MEBO^®^-treated wounds. 

In all samples, the epidermal healing was at an advanced degree at the end of the experiment on day 14. The application of MEBO^®^ ointment somewhat improved the healing process for the wounded animals. However, LP treatment led to obvious improvement of the healing process by inducing full re-epithelization, angiogenesis, dermal restructuring with enhanced collagen deposition, conspicuous fibroblasts, and the development of new hair follicles. 

In the first seven days, the inflammatory phase predominated in the control group, whereas in MEBO^®^- or LP-treated wounds, the proliferative phase, which included decreased inflammation, deposition of collagen, re-epithelialization, and hair follicle development, predominated. LP-treated groups gave the best results of acceleration of wound healing through angiogenesis, epidermal/dermal regeneration, and granulation tissue thickness, and so LP may help decrease inflammation. Behm et al. reported that macrophages, vascular endothelial lining, and probably fibroblasts are cell types that produce many chemokines, cytokines, and growth factors in wound healing that mediate cell migration into the injured tissue, which in turn initiate granulation tissue formation [46].

This enhanced TGF-β gene expression is pivotal, as TGF-β is the critical growth factor with the dominant activity during different wound healing stages. Recruitment and activation of the inflammatory cells (neutrophils and macrophages) are mediated by TGF-β to start the inflammatory phase. Furthermore, the role of TGF-β in the proliferative phase is to orchestrate several cellular actions, including re-epithelialization, angiogenesis, formation of granulation tissue, and extracellular matrix deposition. Moreover, fibroblast proliferation and differentiation into myofibroblasts are stimulated by TGF-β, an event that is crucial in wound contraction in the remodeling phase [47]. Additionally, studies have stated that loss of TGF-β signaling is a common feature in chronic, nonhealing wounds [12,14,48]. These reports support the above-mentioned observations, which showed that LP extract promotes TGF-β expression, suggesting that the wound healing activity of LP is accomplished by increasing TGF-β expression.

Previously, it has been reported that inflammation is an essential process, as it partakes in fighting infection, removing debris, and triggering the proliferation phase. Several proinflammatory mediators are secreted by infiltrating platelets, macrophages, and fibroblasts throughout the inflammatory stage, including TNF-α and IL-1β. These proinflammatory mediators collaborate at the wound site to initiate serious cellular events, such as cell migration, proliferation, ECM protein production, and turnover, to induce tissue regeneration and wound repair [49]. Nevertheless, prolonged inflammation is also harmful, as it can lead to significant cytokines release and increased cell accumulation, resulting in tissue damage, larger scar formation, and, hence, delayed wound healing. Therefore, controlled inflammation and adequate levels of inflammatory cytokines are pivotal for wound healing [50]. This explains the above-mentioned observation that reducing the levels of inflammatory cytokines (TNF-α and IL-1β) by LP extract accelerates the transition from inflammatory to anti-inflammatory responses, and therefore avoids impaired wound healing.

Antioxidants are supposed to hasten wound healing by alleviating wound oxidative stress. They are essential in controlling the harm that ROS may cause to biological components such as DNA, protein, and lipids [7]. LP extract significantly diminished the formation of H_2_O_2_ radical (IC_50_ = 171.6 μg/mL) and scavenged the superoxide radical (IC_50_ of 286.7 µg/mL), indicating antioxidant potential in a dose-dependent manner.

Wound-healing agents are generally categorized as agents that can stimulate fibroblast proliferation, induce keratinocyte cell proliferation and differentiation, and enhance collagen production, or as agents that have antioxidant and anti-inflammatory properties. The presence of two or more of these biological features in an agent suggests that the agent has the potential to be a useful wound-healing agent [51]. So, the above-mentioned antioxidant and anti-inflammatory activities, along with the stimulating fibroblast proliferation and collagen production effect, suggest that LP would be a potential wound-healing agent.

Plant secondary metabolites are gaining popularity these days due to their therapeutic potential through their antioxidant and wound healing properties [7,52]. Natural extracts are commonly composed of hundreds to thousands of metabolites, and the synergism between different metabolites that occurs in natural extracts can be used to describe their bioactivity. Because of the complex chemistry of crude natural extract, it is not usually possible to isolate each compound. Profiling via liquid chromatography/high-resolution mass (LC-HRMS) was used to characterize different metabolites that might be intermediates in the antioxidant and wound healing activities of *Launaea procumbens* methanolic extract.

A molecular docking study was carried out on 16 identified compounds against five wound-healing targets. The first is collagen α-1 (I), the most abundant collagen found in connective tissues, including skin. The second is vascular endothelial growth factor (VEGF), the part of the system that restores the oxygen supply to tissues when blood circulation is inadequate. The third is TNF-α, which regulates the growth and differentiation of a wide variety of cell types. The fourth is TGF-β, a multifunctional set of peptides that controls proliferation, differentiation, and other functions in many cell types. The fifth is IL-1β, formed mainly by the macrophage and which helps the lymphocyte fight infections. Luteolin 8-C-glucoside (orientin) exceptionally demonstrated binding potential to four out of the five investigated proteins and was of special interest for further analysis, posing it as a potential wound-healing drug lead. These results are consistent with previously published ones which confirmed that luteolin 8-C-glucoside is a therapeutic bioactive agent for wound healing by enhancing fibroblast proliferation and migration [53]. Future research is required to analyze luteolin 8-C-glucoside experimentally using a biological model to validate the current results and to prove the hypotheses.

Finally, the high scores and strikingly similar interaction patterns of several ligands in LP methanolic extract with the mentioned wound-healing targets provide some molecular rationale for the wound healing activity of the extract.

## 5. Conclusions

The current study revealed that *Launaea procumbens* extract has a promising wound-healing activity via improving the wound contraction, epithelialization, and modulation of inflammatory mediators. Furthermore, the confirmed H_2_O_2_ and superoxide radical scavenging potential enhanced the wound-healing activity of LP extract. A molecular docking study was also carried out to gain insight into the molecular target proteins collagen α-1, VEGF, TGF-β, TNF-α, and IL-1β, which may be involved in the mechanism of action of the investigated extract. Among the dereplicated compounds, luteolin 8-C-glucoside demonstrated binding potential to four out of five investigated proteins (VEGF, IL-1β, and TNF-α) and is of particular interest for future analysis, posing it as a potential wound-healing drug lead. Hence, *Launaea procumbens* extract can be considered a promising therapy to accelerate wound healing in excisional wounds. The pharmacologically approved activity of chemically characterized extract of *Launaea procumbens* could serve as the base of introducing it as a formula after clinical trials to treat wound-healing problems.

## Data Availability

Data are contained within the article.

## Supplementary Materials

The following supporting information can be downloaded at: https://www.mdpi.com/article/10.3390/antiox11112258/s1, Table S1: The primer sequences of studied genes; Table S2: Annotation of compounds from the methanolic extract of *Launaea procumbens*; Tables S3–S7: In silico docking study results of dereplicated compounds from *Launaea procumbens* within the active site of Collagen α-1 (I) chain, VEGF, TNF-α, TGF-β, and IL-1β. References [54,55,56,57,58,59,60,61,62,63,64,65,66,67,68,69] are cited in Supplementary Materials.

## References

[1] Soni R., Srivastava D. (2017). Wound Repair and Regenerating Effect of Eugenol Isolated from Ethyl Acetate Soluble Fraction of Ethanolic Extract of Cinnamomum tamala Leaves in STZ Diabetic Rats. J. Drug Deliv. Ther..

[2] Carvalho A.R., Diniz R.M., Suarez M.A., Figueiredo C.S., Zagmignan A., Grisotto M.A., Fernandes E.S., da Silva L.C. (2018). Use of some asteraceae plants for the treatment of wounds: From ethnopharmacological studies to scientific evidences. Front. Pharmacol..

[3] Valizadeh R., Hemmati A.A., Houshmand G., Bayat S., Bahadoram M. (2015). Wound healing potential of *Althaea officinalis* flower mucilage in rabbit full thickness wounds. Asian Pac. J. Trop. Biomed..

[4] Hussey M., Bagg M. (2011). Principles of wound closure. Oper. Tech. Sports Med..

[5] Khalil H., Cullen M., Chambers H., Carroll M., Walker J. (2015). Elements affecting wound healing time: An evidence based analysis. Wound Repair Regen..

[6] Cano Sanchez M., Lancel S., Boulanger E., Neviere R. (2018). Targeting oxidative stress and mitochondrial dysfunction in the treatment of impaired wound healing: A systematic review. Antioxidants.

[7] Barku V.Y. (2019). Wound healing: Contributions from plant secondary metabolite antioxidants. Wound Healing-Current Perspectives.

[8] Musa A., Shady N.H., Ahmed S.R., Alnusaire T.S., Sayed A.M., Alowaiesh B.F., Sabouni I., Al-Sanea M.M., Mostafa E.M., Youssif K.A. (2021). Antiulcer potential of *Olea europea* L. Cv. arbequina leaf extract supported by metabolic profiling and molecular docking. Antioxidants.

[9] Siwik D.A., Pagano P.J., Colucci W.S. (2001). Oxidative stress regulates collagen synthesis and matrix metalloproteinase activity in cardiac fibroblasts. Am. J. Physiol.-Cell Physiol..

[10] Rodriguez-Menocal L., Shareef S., Salgado M., Shabbir A., Van Badiavas E. (2015). Role of whole bone marrow, whole bone marrow cultured cells, and mesenchymal stem cells in chronic wound healing. Stem Cell Res. Ther..

[11] Singh K., Agrawal N.K., Gupta S.K., Sinha P., Singh K. (2016). Increased expression of TLR9 associated with pro-inflammatory S100A8 and IL-8 in diabetic wounds could lead to unresolved inflammation in type 2 diabetes mellitus (T2DM) cases with impaired wound healing. J. Diabetes Complicat..

[12] Penn J.W., Grobbelaar A.O., Rolfe K.J. (2012). The role of the TGF-β family in wound healing, burns and scarring: A review. Int. J. Burns Trauma.

[13] Nazmy M.H., Abu-baih D.H., El-Rehany M.A.-A., Fathy M. (2021). Pathways of triple negative breast cancer. Minia J. Med. Res..

[14] Pastar I., Stojadinovic O., Krzyzanowska A., Barrientos S., Stuelten C., Zimmerman K., Blumenberg M., Brem H., Tomic-Canic M. (2010). Attenuation of the transforming growth factor β-signaling pathway in chronic venous ulcers. Mol. Med..

[15] Busilacchi E.M., Costantini A., Mancini G., Tossetta G., Olivieri J., Poloni A., Viola N., Butini L., Campanati A., Goteri G. (2020). Nilotinib Treatment of Patients Affected by Chronic Graft-versus-Host Disease Reduces Collagen Production and Skin Fibrosis by Downmodulating the TGF-β and p-SMAD Pathway. Biol. Blood Marrow Transplant..

[16] Larouche J., Sheoran S., Maruyama K., Martino M.M. (2018). Immune regulation of skin wound healing: Mechanisms and novel therapeutic targets. Adv. Wound Care.

[17] Nosenko M., Ambaryan S., Drutskaya M. (2019). Proinflammatory cytokines and skin wound healing in mice. Mol. Biol..

[18] Yadav E., Singh D., Yadav P., Verma A. (2017). Attenuation of dermal wounds via downregulating oxidative stress and inflammatory markers by protocatechuic acid rich n-butanol fraction of *Trianthema portulacastrum* Linn. in wistar albino rats. Biomed. Pharmacother..

[19] Firdous S.M., Sautya D. (2018). Medicinal plants with wound healing potential. Bangladesh J. Pharmacol..

[20] Shedoeva A., Leavesley D., Upton Z., Fan C. (2019). Wound healing and the use of medicinal plants. Evid.-Based Complement. Altern. Med..

[21] Cheriti A., Belboukhari M., Belboukhari N., Djeradi H. (2012). Phytochemical and biological studies on *Launaea* Cass. genus (Asteraceae) from Algerian Sahara. Phytochemistry.

[22] Asif M., Saadullah M., Yaseen H.S., Saleem M., Yousaf H.M., Khan I.U., Yaseen M., Shams M.U. (2020). Evaluation of In Vivo anti-inflammatory and anti-angiogenic attributes of methanolic extract of *Launaea spinosa*. Inflammopharmacology.

[23] Kumar V., Ghildiyal S., Sherkhane R., Nesari T.M. (2020). Gojihva (*Launaea nudicaulis* [L.] Hook. f.), a potential herb for chronic wound healing: A case study. J. Ayurveda Case Rep..

[24] Rawat P., Saroj L.M., Kumar A., Singh T.D., Tewari S.K., Pal M. (2016). Phytochemicals and cytotoxicity of *Launaea procumbens* on human cancer cell lines. Pharmacogn. Mag..

[25] Wazi S.M., Saima S., Dasti A.A., Subhan S. (2007). Ethanobotnical importance of Salt range species of District Karak, Pakistan. Pak. J. Plant Sci..

[26] Ahmad M., Khan A.M., Manzoor S., Zafar M., Sultana S. (2006). Check list of Medicinal Flora of Tehsil Isakhel. District Mianwali, Pakistan. Ethnobot. Leafl..

[27] Parekh J., Chanda S. (2006). In-Vitro antimicrobial activities of extracts of *Launaea procumbens* roxb. (Labiateae), *Vitis vinifera* L.(Vitaceae) and *Cyperus rotundus* L.(Cyperaceae). Afr. J. Biomed. Res..

[28] Makasana A., Ranpariya V., Desai D., Mendpara J., Parekh V. (2014). Evaluation for the anti-urolithiatic activity of *Launaea procumbens* against ethylene glycol-induced renal calculi in rats. Toxicol. Rep..

[29] Khan R.A., Khan M.R., Sahreen S. (2010). Evaluation of *Launaea procumbens* use in renal disorders: A rat model. J. Ethnopharmacol..

[30] Khan R.A., Khan M.R., Sahreen S., Ahmed M. (2012). Assessment of flavonoids contents and in vitro antioxidant activity of *Launaea procumbens*. Chem. Cent. J..

[31] Reddy M.N., Mishra G.J. (2012). Preliminary phytochemical screening and antibacterial analysis of the leaf extracts of *Launaea procumbens* Roxb. Int. J. Phytopharm..

[32] Tramontina V.A., Machado M.A.N., Filho G.d.R.N., Kim S.H., Vizzioli M.R., Toledo S. (2002). Effect of bismuth subgallate (local hemostatic agent) on wound healing in rats. Histological and histometric findings. Braz. Dent. J..

[33] Sandhu S.K., Kumar S., Raut J., Singh M., Kaur S., Sharma G., Roldan T.L., Trehan S., Holloway J., Wahler G. (2021). Systematic Development and Characterization of Novel, High Drug-Loaded, Photostable, Curcumin Solid Lipid Nanoparticle Hydrogel for Wound Healing. Antioxidants.

[34] Alsenani F., Ashour A.M., Alzubaidi M.A., Azmy A.F., Hetta M.H., Abu-Baih D.H., Elrehany M.A., Zayed A., Sayed A.M., Abdelmohsen U.R. (2021). Wound Healing Metabolites from Peters’ Elephant-Nose Fish Oil: An In Vivo Investigation Supported by In Vitro and In Silico Studies. Mar. Drugs.

[35] Fischer A.H., Jacobson K.A., Rose J., Zeller R. (2008). Hematoxylin and eosin staining of tissue and cell sections. Cold Spring Harb. Protoc..

[36] Boesenberg-Smith K.A., Pessarakli M.M., Wolk D.M. (2012). Assessment of DNA yield and purity: An overlooked detail of PCR troubleshooting. Clin. Microbiol. Newsl..

[37] Livak K.J., Schmittgen T.D. (2001). Analysis of relative gene expression data using real-time quantitative PCR and the 2^−ΔΔCT^ method. Methods.

[38] Hassan H., Abdel-Aziz A. (2010). Evaluation of free radical-scavenging and anti-oxidant properties of black berry against fluoride toxicity in rats. Food Chem. Toxicol..

[39] Sreenivasan S., Ibrahim D., Mohd Kassim M.J.N. (2007). Free radical Scavenging Activity and Total Phenolic Compounds of *Gracilaria changii*. Int. J. Nat. Eng. Sci..

[40] Haggag E.G., Elshamy A.M., Rabeh M.A., Gabr N.M., Salem M., Youssif K.A., Samir A., Muhsinah A.B., Alsayari A., Abdelmohsen U.R. (2019). Antiviral potential of green synthesized silver nanoparticles of *Lampranthus coccineus* and *Malephora lutea*. Int. J. Nanomed..

[41] Berman H.M., Westbrook J., Feng Z., Gilliland G., Bhat T.N., Weissig H., Shindyalov I.N., Bourne P.E. (2000). The protein data bank. Nucleic Acids Res..

[42] Morris G.M., Huey R., Lindstrom W., Sanner M.F., Belew R.K., Goodsell D.S., Olson A.J. (2009). AutoDock4 and AutoDockTools4: Automated docking with selective receptor flexibility. J. Comput. Chem..

[43] Trott O., Olson A.J. (2010). AutoDock Vina: Improving the speed and accuracy of docking with a new scoring function, efficient optimization, and multithreading. J. Comput. Chem..

[44] Jaghoori M.M., Bleijlevens B., Olabarriaga S.D. (2016). 1001 Ways to run AutoDock Vina for virtual screening. J. Comput.-Aided Mol. Des..

[45] Boakye Y.D., Agyare C., Ayande G.P., Titiloye N., Asiamah E.A., Danquah K.O. (2018). Assessment of wound-healing properties of medicinal plants: The case of *Phyllanthus muellerianus*. Front. Pharmacol..

[46] Behm B., Babilas P., Landthaler M., Schreml S. (2012). Cytokines, chemokines and growth factors in wound healing. J. Eur. Acad. Dermatol. Venereol..

[47] Mazumdar S., Ghosh A.K., Dinda M., Das A.K., Das S., Jana K., Karmakar P. (2021). Evaluation of wound healing activity of ethanol extract of *Annona reticulata* L. leaf both In Vitro and in diabetic mice model. J. Tradit. Complement. Med..

[48] Li J., Chou H., Li L., Li H., Cui Z. (2020). Wound healing activity of neferine in experimental diabetic rats through the inhibition of inflammatory cytokines and nrf-2 pathway. Artif. Cells Nanomed. Biotechnol..

[49] Haroon Z.A., Amin K., Saito W., Wilson W., Greenberg C.S., Dewhirst M.W. (2002). SU5416 delays wound healing through inhibition of TGF-β activation. Cancer Biol. Ther..

[50] Chen X., Thibeault S.L. (2010). Role of tumor necrosis factor–α in wound repair in human vocal fold fibroblasts. Laryngoscope.

[51] Houghton P.J., Hylands P.J., Mensah A.Y., Hensel A., Deters A.M. (2005). In vitro tests and ethnopharmacological investigations: Wound healing as an example. J. Ethnopharmacol..

[52] Tsala D.E., Amadou D., Habtemariam S. (2013). Natural wound healing and bioactive natural products. Phytopharmacology.

[53] Che Zain M.S., Lee S.Y., Sarian M.N., Fakurazi S., Shaari K. (2020). In vitro wound healing potential of flavonoid *c*-glycosides from oil palm (*Elaeis guineensis* Jacq.) leaves on 3t3 fibroblast cells. Antioxidants.

[54] Bhukya B., Alam S., Chaturvedi V., Trivedi P., Kumar S., Khan F., Negi A.S., Srivastava S.K. (2021). Brevifoliol and its Analogs: A New Class of Anti-tubercular Agents. Current Topics in Medicinal Chemistry. Curr. Top Med. Chem..

[55] Tanahashi T., Shimada A., Kai M., Nagakura N., Inoue K., Chen C.-C. (1996). An iridoid glucoside from Jasminum hemsleyi. J. Nat. Prod..

[56] Youssif K., Elshamy A., Rabeh M., Gabr N., Haggag E. (2019). A Phytochemical and Biological Review on Plants of The family Aizoaceae. J. Adv. Pharm. Res..

[57] Buszewski B., Kawka S., Suprynowicz Z., Wolski T. (1993). Simultaneous isolation of rutin and esculin from plant material and drugs using solid-phase extraction. J. Pharm. Biomed. Anal..

[58] Kujala T., Loponen J., Pihlaja K. (2001). Betalains and phenolics in red beetroot (*Beta vulgaris*) peel extracts: Extraction and characterisation. Z. Nat. C.

[59] Toyama D.O., Ferreira M.J., Romoff P., Fávero O.A., Gaeta H.H., Toyama M.H. (2014). Effect of chlorogenic acid (5-caffeoylquinic acid) isolated from Baccharis oxyodonta on the structure and pharmacological activities of secretory phospholipase A2 from Crotalus durissus terrificus. BioMed Res. Int..

[60] Croteau R., Ketchum R.E., Long R.M., Kaspera R., Wildung M.R. (2006). Taxol biosynthesis and molecular genetics. Phytochem. Rev..

[61] Pan R.-L., Chen D.-H., Si J.-Y., Zhao X.-H. (2007). Cimifoetisides VI and VII Two new cyclolanostanol triterpene glycosides from the aerial parts of Cimicifuga foetida. J. Asian Nat. Prod. Res..

[62] Piattelli M., Impellizzeri G. (1970). 2-Descarboxybetanidin, a minor betacyanin from Carpobrotus acinaciformis. Phytochemistry.

[63] Zeng X., Tian J., Cui L., Wang Y., Su Y., Zhou X., He X. (2014). The phenolics from the roots of Livistona chinensis show antioxidative and obsteoblast differentiation promoting activity. Molecules.

[64] Li X.-H., Hui Y.-H., Rupprecht J., Liu Y.-M., Wood K., Smith D., Chang C.-J., McLaughlin J. (1990). Bullatacin, bullatacinone, and squamone, a new bioactive acetogenin, from the bark of Annona squamosa. J. Nat. Prod..

[65] Hou C., Brown W., Labeda D., Abbott T., Weisleder D. (1997). Microbial production of a novel trihydroxy unsaturated fatty acid from linoleic acid. J. Ind. Microbiol. Biotechnol..

[66] Almahy H.A., Fouda H.A.-R. (2013). Isolation of luteolin 8-C-β-D-glucopyranoside from the roots of Salvadora persica (RUTACEAE). J. Curr. Chem. Pharm. Sci..

[67] Echavarría A.P., D’Armas H., Matute N., Cano J.A. (2020). Phytochemical analyses of eight plants from two provinces of Ecuador by GC-MS. Int. J. Herb. Med..

[68] Táborská E., Bochořáková H., Soušek J., Sedmera P., Havlíček V., Šimánek V. (1997). Fumaflorine, a new 1-benzylisoquinoline alkaloid from Fumaria densiflora. Heterocycles.

[69] Raab T., Barron D., Vera F.A., Crespy V., Oliveira M., Williamson G. (2010). Catechin glucosides: Occurrence, synthesis, and stability. J. Agric. Food Chem..

