# Supplementary material for: Wound Healing and Antioxidant Properties of Launaea procumbens Supported by Metabolomic Profiling and Molecular Docking
Source: Antioxidants (Basel). 2022 Nov 16;11(11):2258. doi: 10.3390/antiox11112258 (PMC9687060; doi:10.3390/antiox11112258)
Supplement: Supplementary file 1 [file antioxidants-11-02258-s001.zip › antioxidants-1991439-supplementary.pdf]

SUPPLEMENTARY DATA

Wound healing and antioxidant properties of *Launaea procumbens* supported by metabolomic profiling and molecular docking

Shaimaa R. Ahmed[1,2*], Ehab M. Mostafa[1,3], Arafa Musa[1,3], Enas Ezzat Rateb[4], Mohammad M. Al-Sanea[5], Dalia H. Abu-Baih[6], Mahmoud A. Elrehany[6,7], Entesar Ali Saber[8], Mostafa E. Rateb[9], Usama Ramadan Abdelmohsen[10,11]

[1]Department of Pharmacognosy, College of Pharmacy, Jouf University, Sakaka, Aljouf 72341, Saudi Arabia; srmorsi@ju.edu.sa

[2]Department of Pharmacognosy, Faculty of Pharmacy, Cairo University, Kasr el-Aini Street, Cairo 11562, Egypt; shaimaa.ahmed@pharma.cu.edu.eg

[3]Pharmacognosy and Medicinal Plants Department, Faculty of Pharmacy (Boys), Al-Azhar University, Cairo, 11884, Egypt

[4] Department of Physiology, Faculty of Medicine, Beni-Suef University, Beni-Suef 62521, Egypt, enas_omyahya@med.bsu.edu.eg.

[5]Pharmaceutical Chemistry Department, College of Pharmacy, Jouf University, Sakaka, Aljouf 72341, Saudi Arabia; mmalsanea@ju.edu.sa

[6] Department of Biochemistry and molecular biology, Faculty of Pharmacy, Deraya University, New Minia City, Minia 61111, Egypt; Dalia.hamdy@deraya.edu.eg (D.H.A.-B.); Mahmoud.elrehany@deraya.edu.eg (M.A.E.)

[7] Department of Biochemistry, Faculty of Medicine, Minia University, Minia 61519, Egypt

[8] Department of Medical sciences (Histology), Deraya University, New Minia City, Minia 61111, Egypt

[9] School of Computing, Engineering & Physical Sciences, University of the West of Scotland, Paisley PA1 2BE, UK; Mostafa.Rateb@uws.ac.uk

[10] Department of Pharmacognosy, Faculty of Pharmacy, Deraya University, Universities Zone, New Minia City, Minia, Egypt

[11] Department of Pharmacognosy, Faculty of Pharmacy, Minia University, Minia 61519, Egypt; usama.ramadan@mu.edu.eg

* Correspondence: Department of Pharmacognosy, College of Pharmacy, Jouf University, Sakaka, Aljouf 72341, Saudi Arabia; srmorsi@ju.edu.sa

Abstract: Wounds adversely affect people's quality of life and have psychological, social, and economic impacts. Ayurvedic and herbal remedies of *Launaea procumbens* (LP) are used to treat wounds. Excision wound modelling was executed to evaluate the wound healing promoting activity of LP extract in rabbits. The parameters studied were the percentage of wound closure, histopathological study, and gene expression analysis. *In vitro* antioxidant activity was evaluated using hydrogen peroxide (H_2O_2) and superoxide radical scavenging assays. In addition, LP metabolite profiling was performed using LC-HRMS, and the identified compounds were docked against important wound healing targets including vascular endothelial growth factor (VEGF), collagen α-1, tumor necrosis factor-α (TNF-α), interleukin-1β (IL-1β) and transforming growth factor-β (TGF-β). Topical application of LP significantly promoted wound closure ($p<0.001$). Histological analysis revealed a considerable rise in the number of fibroblasts, the amount of collagen, and its cross-linking in LP-treated wounds. Gene expression patterns showed significant elevation of TGF-β levels and down regulation of the inflammatory TNF-α, and IL-1β levels in LP-treated wounds. In a dose-dependent manner, LP extract significantly diminished the formation of H_2O_2 radical and scavenged the superoxide radical, indicating antioxidant potential. Dereplication of the secondary metabolites resulted in the annotation of 16 metabolites. Among dereplicated compounds, luteolin 8-C-glucoside demonstrated binding potential to four investigated targets (VEGF, interleukin 1 β, TNF-α, and collagen α-1). To conclude, *Launaea procumbens* extract could be regarded as a promising topical therapy to promote wound healing in excisional wounds, and luteolin 8-C-glucoside, one of its constituents, is a potential wound healing drug lead.

Keywords: *Launaea procumbens*; antioxidant; wound healing; LC-HRMS profiling; docking; Luteolin 8-C-glucoside

Table S1. The primer sequences of studied genes

Gene name	GenBank accession		
IL- β1	NC_013670.1	Forward	5'-AGCTTCTCCAGAGCCACAAC-3'
		Reverse	5'-CCTGACTACCCTCACGCACC-3'
GAPDH	NC_013676.1	Forward	5'-GTCAAGGCTGAGAACGGGAA-3'
		Reverse	5'-ACAAGAGAGTTGGCTGGGTG-3'
TGF-β1	NC_013672.1	Forward	5'-GACTGTGCGTTTTGGGTTCC-3'
		Reverse	5'-CCTGGGCTCCTCCTAGAGTT-3'
TNF-α	NC_013680.1	Forward	5'-GAGAACCCCACGGCTAGATG-3'
		Reverse	5'-TTCTCCAACTGGAAGACGCC-3'

Table S2 Annotation of compounds from the methanolic extract of *Launaea procambens*

Number	M/Z	RT (min.)	M.wt.	Name	Molecular formula	Chemical class	References
1	555.25847	0.3877	556.26574	11(15→1)-Abeo-4(20),11-taxadiene-5,7,9,10,13,15-hexol; (5α,7β,9α,10β,13α)-form, 10-Benzoyl, 7,9-di-Ac	$C_{31}H_{40}O_9$	Tetracyclic diterpenoid	1
2	717.29723	1.2599	718.3045	Loganic acid; 7-O-(6R-β-D-Glucopyranosyloxy-2,6-dimethyl-2E,7-octadienoyl), Me ester	$C_{33}H_{50}O_{17}$	Iridoid glycoside	2
3	325.24573	1.7451556	326.25301	Touruosamine	$C_{20}H_{26}N_2O_2$	Alkaloid	3
4	339.16009	1.8050278	340.16736	Esculin	$C_{15}H_{16}O_9$	Coumarin glucoside	4
5	338.15887	2.1028167	339.16615	Vulgaxanthin I	$C_{14}H_{17}N_3O_7$	Betaxanthins	5
6	353.13792	2.1120222	354.1452	5-caffeoylquinic acid (Chlorogenic acid)	$C_{16}H_{18}O_9$	Phenolic acid	6
7	304.24608	2.1133056	305.25335	3`-methoxy-4`-o-methyljoubertiaminol	$C_{18}H_{27}NO_3$	Alkaloid	7
8	649.39398	2.2638222	650.40125	Cimigenol; 3-*O*-β-D-Galactopyranoside	$C_{36}H_{58}O_{10}$	Triterpenoid	8
9	387.12881	2.3517944	388.13609	Isobetanidin	$C_{18}H_{16}N_2O_8$	Indolecarboxylic acid	9
10	607.529	2.3523778	608.53627	Glycerol 1-alkanoates; Glycerol 1-(12-hydroxy-13E,15E-pentatriacontanoate)	$C_{38}H_{72}O_5$	Fatty acid ester	10
11	621.47308	2.4930833	622.48036	Bullatacinone	$C_{37}H_{66}O_7$	Lactone	11
12	329.34775	4.0174333	330.35503	11,12,13-Trihydroxy-9-octadecenoic acid	$C_{18}H_{34}O_5$		12
13	446.0207	4.5714889	447.02798	Luteolin 8-C-glucoside	$C_{21}H_{19}O_{11}$	Flavonoid glycoside	13
14	295.43085	5.5372722	296.43813	Phytol	$C_{20}H_{40}O$	acyclic diterpene alcohol	14
15	394.09377	5.5884833	395.10105	Fumaflorine; Me ester	$C_{21}H_{17}NO_7$	Alkaloid	15
16	451.06864	5.9696222	452.07591	Catechin-5-*O*-glucoside	$C_{21}H_{24}O_{11}$	Phenolic compound	16

Table S3. *In silico* docking study results of dereplicated compounds from *Launaea procumbens* within the active site of Collagen α-1 (I) chain (PDB ID1Q7D)

**Compounds**	**Score (kcal/mol)**
Luteolin 8-C-glucoside	-6.7
Loganicacid_7-O-(6R-ß-D-Glucopyranosyloxy-2,6-dimethyl-2E,7-octadienoyl),Meester	-5.9
Isobetanidine	-5.9
Cimigenol_3-O-ß-D-Galactopyranoside	-5.9
Catechin-5-O-glucoside	-5.8
Esculin	-5.6
Fumaflorine_Meester	-5.6
Chlorogenicacid	-5.4
VulgaxanthinI	-5.3
Bullatacinone	-5.3
Touruosamine	-5.0
11,12,13-Trihydroxy-9-octadecenoic acid	-4.8
11(151)-Abeo-4(20),11-taxadiene-5,7,9,10,13,15-hexol_(5alfa,7beta,9alfa,10beta,13alfa)-form,10-Benzoyl,7,9-di-Ac	-4.7
Phytol	-4.6
Glycerol1-alkanoates_Glycerol1-(12-hydroxy-13E,15E-pentatriacontanoate)	-4.1

Table S4. *In silico* docking study results of dereplicated compounds from *Launaea procumbens* within the active sites of VEGF (PDB ID 1FLT)

**Compounds**	**Docking Score (kcal/mol)**
Loganicacid_7-O-(6R-ß-D-Glucopyranosyloxy-2,6-dimethyl-2E,7-octadienoyl),Meester	-8.1
Luteolin 8-C-glucoside	-7.8
Catechin-5-O-glucoside	-7.6
Chlorogenicacid	-7.4
Esculin	-7.3
Isobetanidine	-7.2
Cimigenol_3-O-ß-D-Galactopyranoside	-7.2
Fumaflorine_Meester	-7.1
Touruosamine	-6.9
11(151)-Abeo-4(20),11-taxadiene-5,7,9,10,13,15-hexol_(5alfa,7beta,9alfa,10beta,13alfa)-form,10-Benzoyl,7,9-di-Ac	-6.9
VulgaxanthinI	-6.7
Bullatacinone	-6.6
11,12,13-Trihydroxy-9-octadecenoic acid	-5.5
Phytol	-5.2
Glycerol1-alkanoates_Glycerol1-(12-hydroxy-13E,15E-pentatriacontanoate)	-4.8

Table S5. *In silico* docking study results of dereplicated compounds from *Launaea procumbens* within the active site of TNF-α (PDB ID: 2AZ5)

**Compounds**	**Docking Score (kcal/mol)**
2AZ5 co-crystallized ligand	-9.0
Luteolin 8-C-glucoside	-8.5
11(151)-Abeo-4(20),11-taxadiene-5,7,9,10,13,15-hexol_(5alfa,7beta,9alfa,10beta,13alfa)-form,10-Benzoyl,7,9-di-Ac	-8.5
Cimigenol_3-O-ß-D-Galactopyranoside	-8.5
Fumaflorine_Meester	-8.2
Catechin-5-O-glucoside	-7.5
Loganicacid_7-O-(6R-ß-D-Glucopyranosyloxy-2,6-dimethyl-2E,7-octadienoyl),Meester	-7.4
Isobetanidine	-7.4
Esculin	-7.2
Touruosamine	-7.1
Chlorogenicacid	-6.8
VulgaxanthinI	-6.6
11,12,13-Trihydroxy-9-octadecenoic acid	-6.2
Bullatacinone	-6.0
Phytol	-5.8
Glycerol1-alkanoates_Glycerol1-(12-hydroxy-13E,15E-pentatriacontanoate)	-5.7

Table S6. *In silico* docking study results of dereplicated compounds from *Launaea procumbens* within the active site of TGF-β domain (PDB ID: 6B8Y)

**Compounds**	**Docking Score (kcal/mol)**
6B8Y co-crystallized ligand	-12.2
Catechin-5-O-glucoside	-10.2
Luteolin 8-C-glucoside	-9.5
Isobetanidine	-8.8
Fumaflorine_Meester	-8.7
Chlorogenicacid	-8.7
VulgaxanthinI	-8.3
Esculin	-8.3
Bullatacinone	-7.7
Touruosamine	-7.4
Phytol	-7.2
Glycerol1-alkanoates_Glycerol1-(12-hydroxy-13E,15E-pentatriacontanoate)	-6.5
Loganicacid_7-O-(6R-ß-D-Glucopyranosyloxy-2,6-dimethyl-2E,7-octadienoyl),Meester	-6.3
11,12,13-Trihydroxy-9-octadecenoic acid	-6.0
11(151)-Abeo-4(20),11-taxadiene-5,7,9,10,13,15-hexol_(5alfa,7beta,9alfa,10beta,13alfa)-form,10-Benzoyl,7,9-di-Ac	-2.2
Cimigenol_3-O-ß-D-Galactopyranoside	-1.6

Table S7. *In silico* docking study results of dereplicated compounds from *Launaea procumbens* within the active site of IL-1β (PDB ID: 6Y8M)

**Compounds**	**Docking Score (kcal/mol)**
Luteolin 8-C-glucoside	-6.4
11(151)-Abeo-4(20),11-taxadiene-5,7,9,10,13,15-hexol_(5alfa,7beta,9alfa,10beta,13alfa)-form,10-Benzoyl,7,9-di-Ac	-6.3
Esculin	-6.0
Catechin-5-O-glucoside	-6.0
Isobetanidine	-5.8
Chlorogenicacid	-5.8
Cimigenol_3-O-ß-D-Galactopyranoside	-5.7
Loganicacid_7-O-(6R-ß-D-Glucopyranosyloxy-2,6-dimethyl-2E,7-octadienoyl),Meester	-5.5
Fumaflorine_Meester	-5.4
Touruosamine	-5.1
VulgaxanthinI	-4.9
Bullatacinone	-4.9
6Y8M co-crystallized ligand	-4.8
11,12,13-Trihydroxy-9-octadecenoic acid	-4.6
Glycerol1-alkanoates_Glycerol1-(12-hydroxy-13E,15E-pentatriacontanoate)	-4.0
Phytol	-3.9

References

1. Bhukya, B., Alam, S., Chaturvedi, V., Trivedi, P., Kumar, S., Khan, F., Negi, A.S., Srivastava, S.K., 2020. Brevifoliol and its Analogs: A New Class of Anti-tubercular Agents. Current Topics in Medicinal Chemistry.
2. Tanahashi, T., Shimada, A., Kai, M., Nagakura, N., Inoue, K., Chen, C.-C., 1996. An iridoid glucoside from Jasminum hemsleyi. Journal of natural products 59(8), 798-800.
3. Youssif, K., Elshamy, A., Rabeh, M., Gabr, N., Haggag, E., 2019. A Phytochemical and Biological Review on Plants of The family Aizoaceae. Journal of Advanced Pharmacy Research 3(4), 158-181.
4. Buszewski, B., Kawka, S., Suprynowicz, Z., Wolski, T., 1993. Simultaneous isolation of rutin and esculin from plant material and drugs using solid-phase extraction. Journal of pharmaceutical and biomedical analysis 11(3), 211-215.
5. Kujala, T., Loponen, J., Pihlaja, K., 2001. Betalains and phenolics in red beetroot (Beta vulgaris) peel extracts: extraction and characterisation. Zeitschrift für Naturforschung C 56(5-6), 343-348.
6. Toyama, D.O., Ferreira, M.J., Romoff, P., Fávero, O.A., Gaeta, H.H., Toyama, M.H., 2014. Effect of chlorogenic acid (5-caffeoylquinic acid) isolated from Baccharis oxyodonta on the structure and pharmacological activities of secretory phospholipase A2 from Crotalus durissus terrificus. BioMed research international 2014.
7. Croteau, R., Ketchum, R.E., Long, R.M., Kaspera, R., Wildung, M.R., 2006. Taxol biosynthesis and molecular genetics. Phytochemistry Reviews 5(1), 75-97.
8. Pan, R.-L., Chen, D.-H., Si, J.-Y., Zhao, X.-H., 2007. Cimifoetisides VI and VII Two new cyclolanostanol triterpene glycosides from the aerial parts of Cimicifuga foetida. Journal of Asian natural products research 9(2), 97-102.
9. Piattelli, M., Impellizzeri, G., 1970. 2-Descarboxybetanidin, a minor betacyanin from Carpobrotus acinaciformis. Phytochemistry 9(12), 2553-2556.
10. Zeng, X., Tian, J., Cui, L., Wang, Y., Su, Y., Zhou, X., He, X., 2014. The phenolics from the roots of Livistona chinensis show antioxidative and obsteoblast differentiation promoting activity. Molecules 19(1), 263-278.
11. Li, X.-H., Hui, Y.-H., Rupprecht, J., Liu, Y.-M., Wood, K., Smith, D., Chang, C.-J., McLaughlin, J., 1990. Bullatacin, bullatacinone, and squamone, a new bioactive acetogenin, from the bark of Annona squamosa. Journal of natural products 53(1), 81-86.
12. Hou, C., Brown, W., Labeda, D., Abbott, T., Weisleder, D., 1997. Microbial production of a novel trihydroxy unsaturated fatty acid from linoleic acid. Journal of Industrial Microbiology and Biotechnology 19(1), 34-38.
13. Almahy, H.A., Fouda, H.A.-R., 2013. Isolation of luteolin 8-C-β-D-glucopyranoside from the roots of Salvadora persica (RUTACEAE). Journal of Current Chemical and Pharmaceutical Sciences 3(1), 49-53.
14. Echavarría, A.P., D'Armas, H., Matute, N., Cano, J.A., 2020. Phytochemical analyses of eight plants from two provinces of Ecuador by GC-MS. Int J Herb Med 8, 10-20.
15. Táborská, E., Bochořáková, H., Soušek, J., Sedmera, P., Havlíček, V., Sĭmánek, V.m., 1997. Fumaflorine, a new 1-benzylisoquinoline alkaloid from Fumaria densiflora. Heterocycles 4(45), 817-821.
16. Raab, T., Barron, D., Vera, F.A., Crespy, V., Oliveira, M., Williamson, G., 2010. Catechin glucosides: occurrence, synthesis, and stability. Journal of agricultural and food chemistry 58(4), 2138-2149.
